# Anti-Inflammatory Activity of Marine Ovothiol A in an *In Vitro* Model of Endothelial Dysfunction Induced by Hyperglycemia

**DOI:** 10.1155/2018/2087373

**Published:** 2018-04-19

**Authors:** Immacolata Castellano, Pamela Di Tomo, Natalia Di Pietro, Domitilla Mandatori, Caterina Pipino, Gloria Formoso, Alessandra Napolitano, Anna Palumbo, Assunta Pandolfi

**Affiliations:** ^1^Department of Biology and Evolution of Marine Organisms, Stazione Zoologica Anton Dohrn, Napoli, Italy; ^2^Department of Medicine and Aging Sciences, “G. d'Annunzio” University Chieti-Pescara, Chieti, Italy; ^3^Centro Scienze dell'Invecchiamento-Medicina Traslazionale (CeSI-MeT), “G. d'Annunzio” University Chieti-Pescara, Chieti, Italy; ^4^Department of Medical, Oral and Biotechnological Sciences, “G. d'Annunzio” University Chieti-Pescara, Chieti, Italy; ^5^Department of Chemical Sciences, University of Naples Federico II, Naples, Italy

## Abstract

Chronic hyperglycemia is associated with oxidative stress and vascular inflammation, both leading to endothelial dysfunction and cardiovascular disease that can be weakened by antioxidant/anti-inflammatory molecules in both healthy and diabetic subjects. Among natural molecules, ovothiol A, produced in sea urchin eggs to protect eggs/embryos from the oxidative burst at fertilization and during development, has been receiving increasing interest for its use as an antioxidant. Here, we evaluated the potential antioxidative/anti-inflammatory effect of purified ovothiol A in an *in vitro* cellular model of hyperglycemia-induced endothelial dysfunction employing human umbilical vein endothelial cells (HUVECs) from women affected by gestational diabetes (GD) and from healthy mothers. Ovothiol A was rapidly taken up by both cellular systems, resulting in increased glutathione values in GD-HUVECs, likely due to the formation of reduced ovothiol A. In tumor necrosis factor-*α*-stimulated cells, ovothiol A induced a downregulation of adhesion molecule expression and decrease in monocyte-HUVEC interaction. This was associated with a reduction in reactive oxygen and nitrogen species and an increase in nitric oxide bioavailability. These results point to the potential antiatherogenic properties of the natural antioxidant ovothiol A and support its therapeutic potential in pathologies related to cardiovascular diseases associated with oxidative/inflammatory stress and endothelial dysfunction.

## 1. Introduction

One of the major challenges of the recent research in biomedicine is the discovery of new natural products to develop drugs and dietary supplements that could prevent and relieve pathologies associated with chronic low-grade inflammation and oxidative stress. Among these, diabetes is one of the most widespread. It is associated with oxidative stress and vascular chronic inflammation, alterations underlying the development of cardiovascular disease [1]. In particular, endothelial dysfunction is associated with vascular disease occurrence and is characterized by an increased expression of endothelial adhesion molecules and the recruitment of monocytes to the intima, a pivotal and critical event in promoting atherosclerosis [1, 2]. Nitric oxide (NO), constitutively generated by endothelial cells, plays a key role in the maintenance of vascular homeostasis through the reduction of proinflammatory response that characterizes the early stages of atherosclerosis, especially during chronic hyperglycemia [3]. The preservation of endothelial NO bioavailability, leading to increased vascular cGMP levels, is therefore considered beneficial to endothelial functions and more in general to vascular health. In particular, in vascular smooth muscle cells, the bioavailable NO activates soluble guanylate cyclase (sGC) leading to an increase in cGMP content and subsequent arterial relaxation, thus contributing to the maintenance of vascular homeostasis and health [3].

Notably, although the mechanisms are not fully understood, the consumption of natural molecules with known antioxidative/anti-inflammatory activity has often been associated with decreased cardiovascular risk in healthy and diabetic subjects [4–6]. In the last decades, great efforts have been devoted to the discovery of new active compounds from less explored natural sources, such as marine environments, which offer a greater biodiversity compared to the terrestrial ones, and represent a huge reservoir of bioactive molecules. In this scenario, an increasing interest has recently been focused on ovothiols (*π*-*N*-methyl-5-tiohistidines) isolated from several marine sources, including invertebrates, algae, and protozoa [7]. Three different forms of ovothiols have been characterized (A, B, and C) which differ in the degree of methylation at the nitrogen of the aminoacidic side chain. Ovothiol A, ovothiol B, and ovothiol C correspond to the unmethylated, monomethylated, and dimethylated forms, respectively. Ovothiol A, initially discovered in the eggs of some echinoderms (e.g. the sea urchin *Paracentrotus lividus*) [8, 9] and in the biological fluids of some mollusks and polychaetes [10, 11], was also found in some microalgae and protozoa [12–17]. Ovothiol B was found in the ovaries of the scallop *Chlamys hastata* [18], whereas ovothiol C in the eggs of some sea urchin species [8, 19]. All ovothiols display unusual antioxidant properties, thanks to the peculiar position of the thiol group on the imidazole ring of histidine [20–22]. In particular, ovothiols can play a key role in controlling the cellular redox balance and are maintained in the reduced state in the eggs by reduced glutathione [22, 23]. In sea urchin, the role of ovothiol A has been associated with a detoxification function from peroxides produced during fertilization [24] and in response to environmental stressors during embryo development in sea water [25]. Although a therapeutic use in the relief of pathogenesis related to oxidative stress can be envisaged for this class of molecules, up to date, only the neuroprotective activity of an ovothiol analogue through the regulation of redox homeostasis has been documented [26]. On the other hand, we have recently reported the antiproliferative activity of ovothiol A in hepatocarcinoma cell lines induced via an autophagic mechanism, not involving scavenging of reactive oxygen species (ROS) by the molecule [27].

Lately, we have characterized the phenotype of human umbilical vein endothelial cells obtained from umbilical cords of gestational diabetic mothers (GD-HUVECs) and thus chronically exposed *in vivo* to hyperglycemia and to a proinflammatory environment during pregnancy. As compared to control cells from healthy mothers (C-HUVECs), the GD-HUVECs exhibit durable proatherogenic modifications such as enhanced monocyte adhesion, nitric oxide synthase (NOS) expression and activity, increased superoxide generation together with increased nitrotyrosine levels, and reduced NO bioavailability [28]. Overall, these factors have been identified as molecular modifications of cellular homeostasis eventually impacting on endothelial dysfunction.

In this study, we have examined the potential anti-inflammatory effect of ovothiol A in *in vitro* GD-HUVEC cultures, as a model of endothelial low-grade chronic inflammation. Interestingly, in exploiting the effect of TNF-*α* as a molecule capable of stimulating mechanisms involved in endothelial dysfunction [29, 30], we have found that in TNF-*α*-stimulated GD-HUVECs, ovothiol A induced a downregulation of adhesion molecule expression and monocyte-HUVEC interaction, and this effect was associated with a reduction of nitro oxidative species and an increase in NO bioavailability.

## 2. Materials and Methods

### 2.1. Preparation of Ovothiol A from Sea Urchin *P. lividus* Eggs

Ovothiol A was obtained as disulphide from *P. lividus* eggs by a procedure involving fractionation of the lipid-free aqueous extract of sea urchin eggs by consecutive ion exchange chromatography with HCl at different molarities, with a yield of 2.5 mg ovothiol A/10 g of eggs [27].

### 2.2. Cell Cultures and Experimental Protocols

As previously described [1], umbilical cords were obtained from randomly selected healthy Caucasian control (C, *n* = 12) and with gestational diabetes (GD, *n* = 12) mothers delivering at the Hospital of Chieti and Pescara. In detail, normotensive GD and C women, matched for age and body mass index (BMI), underwent a 75 g 2 h oral glucose tolerance test (OGTT) during the 24–28th gestational weeks (gw) according to the guidelines. Donors' characteristics are described in Table 1. All procedures were in agreement with the ethical standards of the Institutional Committee on Human Experimentation (reference number: 1879/09COET) and with the Declaration of Helsinki principles. After approval of the protocol by the Institutional Review Board, signed informed consent was obtained from each participating subject [1]. Umbilical cords were collected immediately after delivery given in the 36–40th gw, then HUVEC explants were performed to obtain primary C- and GD-HUVECs that were used between the 3rd and 5th passages *in vitro*, as previously reported [31]. Briefly, HUVECs were grown to subconfluence in complete low-glucose (1 g/L) culture medium composed of Dulbecco's Modified Eagle Medium (DMEM, Cat. D6046, Sigma-Aldrich, Saint Louis, USA) and M199 endothelial growth medium (M199, Cat. M4530, Sigma-Aldrich) (ratio 1 : 1) supplemented with 20% fetal bovine serum (FBS, Gibco-Life Technologies, Monza, Italy) and 1% penicillin/streptomycin and 1% L-glutamine (Sigma-Aldrich). Subsequently, the cells were serum-starved and incubated for 16 h with TNF-*α* (Sigma-Aldrich) 1 ng/mL or 10 ng/mL, following a 24 h preincubation with ovothiol A at different concentrations (10–50–100 *μ*M) or with medium alone as basal condition.

All experiments were performed in triplicate using 3 different cell strains each time. In total, all experiments were carried out employing 12 strains of C- and 12 of GD-HUVECs, respectively.

### 2.3. MTT Assay

1 × 10^5^ C- and GD-HUVECs were plated in 96-well plates, grown to subconfluence, and stimulated for 24 h with growing doses of ovothiol A or with the medium alone (as control), in the presence or absence of TNF-*α* stimulation. Cell viability was assessed by the 3-(4,5-dimethylthiazolyl-2)-2,5-diphenyltetrazolium bromide (MTT, Sigma-Aldrich) assay as previously described [31].

### 2.4. Analysis of Ovothiol A in Cell Cultures

HUVEC cells (1.2 × 10^6^ cells) were cultured in flasks (25 cm^2^) in the absence and in the presence of 50 *μ*M ovothiol A and collected after incubation at different time intervals. The culture media were recovered by centrifugation, frozen, and stored at −20°C for ovothiol A quantitation by HPLC analysis.

The cellular pellets were suspended in PBS pH 7.5 and sonicated three times for 15 sec and at 30% amplitude. The samples were then centrifuged at 13,000 rpm over 15 min to remove cellular debris and ultrafiltered by Microcon 3 to remove all molecules with MW > 3 kDa, frozen, and stored at −20°C for ovothiol A quantitation. All experiments were repeated at least three times. HPLC analyses were performed in duplicate on an LC-10AD instrument equipped with binary pumps and a Shimadzu SPD-10AVP detector set at 254 nm and 280 nm. A Phenomenex Synergi SphereClone octadecylsilane (25 cm × 0.46 cm, 4 *μ*m particle size) column was used with 1% formic acid taken to pH 4.5 with ammonia, as the eluant, at a 0.7 mL/min flow rate. Samples exhibiting a peak at the same elution time of standard solutions of ovothiol A were checked by LC/MS run on an LC/MS ESI-TOF 1260/6230DA Agilent instrument operating in positive ionization mode in the following conditions: nebulizer pressure 35 psig; drying gas (nitrogen) 8 L/min, 325°C; capillary voltage 3500 V; and fragmentor voltage 175 V. An Eclipse Plus C18 column, 150 × 4.6 mm, 5 *μ*m, was used at a flow rate of 0.4 mL/min: RT 9 min, 0.1% formic acid, pH 4.5 with ammonia, *m*/*z* 401 ([M + H]+) for disulphide form, and *m*/*z* 202 ([M + H]+) for ovothiol A reduced form.

### 2.5. Determination of Intracellular Glutathione Levels

Total glutathione was determined by using the Glutathione Assay Kit (Sigma). Briefly, an aliquot of ultrafiltered samples was added to 3 volumes of 5% 5-sulfosalicylic acid and mixed. Samples were then frozen (−80°C) and thawed at 37°C twice, left for 5 min at 4°C, and finally centrifuged at 10,000*g* for 10 min. In this procedure, following the incubation with glutathione reductase and NADPH, glutathione was totally recovered in the reduced form and thus determined by monitoring the reduction of 5,5-dithiobis(2-nitrobenzoic acid) (DTNB) to 5-thio-2-nitrobenzoic acid (TNB), at 412 nm by a Thermo Scientific™ Multiskan™ FC Microplate Photometer. The response of this overall assay procedure was also challenged by a solution of ovothiol A in its disulphide form, a solution resulting from incubation of reduced glutathione and ovothiol A disulphide at 1 to 0.5 molar ratio to generate the mixed ovothiol A-glutathione disulphide, and a reference solution with the same reduced glutathione content and devoid of ovothiol A disulphide. These solutions were prepared in water and/or 0.05 M phosphate buffer PBS at pH 7.5.

### 2.6. Monocyte Adhesion Assay

To evaluate the adhesion of monocytes to HUVEC monolayers, U937 cell lines (European Collection of Authenticated Cell Cultures, ECACC, Salisbury, UK) were grown in RPMI medium supplemented with 10% FBS and *β*-mercaptoethanol (1 *μ*M, Sigma-Aldrich). C- and GD-HUVECs were grown to confluence in six-well culture plates and treated as described in the experimental protocol. U937 cell adhesion was evaluated by counting the number of the adherent U937 cells on HUVEC monolayers, as previously described [31]. Furthermore, as negative controls, C- and GD-HUVECs were treated with antibodies against VCAM-1 or ICAM-1 at saturating concentrations (1 *μ*g/1 × 10^6^ cells, Santa Cruz Biotechnology) 1 hour before the assay. For each condition, 6 photos were taken; for each photo, the count was made on 4 randomly selected fields using a predetermined 8-dial grid.

### 2.7. Flow Cytometry

For flow cytometry analysis, C- and GD-HUVECs were cultured in flasks (25 cm^2^) and stimulated as described in the experimental protocols. Non permeabilized cells were treated as previously described [31] and then incubated with an anti-VCAM-1 PE conjugate (1 : 100, phycoerythrin; BioLegend, San Diego, CA, USA) and with an anti-ICAM-1 FITC conjugate (1 : 100, fluorescein isothiocyanate; BioLegend) antibodies, both for 30 min at 4°C.

In order to evaluate the intracellular levels of peroxynitrite (ONOO^−^), 5 × 10^5^ C- and GD-HUVECs were incubated with 10 *μ*M of HKGreen-4A (30 min at 37°C), as described previously [31]. It is a probe with high selectivity and sensitivity for peroxynitrite detection, kindly provided by the laboratory of Professor Dan Yang [32]. Under the same conditions, the intracellular levels of superoxide anion (O_2_^•−^) were evaluated in C- and GD-HUVECs using the probe hydroethidine (5 *μ*M for 30 min at 37°C, Thermo Fisher Scientific). To stimulate NO endogenous production, C- and GD-HUVECs were preincubated with ionomycin (Iono, 50 nM), which activates NOS through the induction of the intracellular levels of calcium (Ca^2+^). To stimulate the endogenous production of peroxynitrite, cells were preincubated (30 min) with phorbol 12-myristate 13-acetate (PMA, 200 ng/mL) in combination with Iono. To stimulate an endogenous production of superoxide anion, cells were treated with H_2_O_2_ (300 *μ*M). All samples were analyzed using a FACSCalibur or FACSCanto II (BD Biosciences, California, USA). The levels of membrane proteins of VCAM-1 and ICAM-1 and fluorescence detection of ONOO^−^ and O_2_^•−^ were evaluated by cytometric analysis of over 10,000 events for each sample. All data were analyzed using FACSDiva (BD Biosciences) and FlowJo v.8.8.6 software (TreeStar, Ashland, OR) and expressed as MFI (mean fluorescence intensity) ratio (signal to noise ratio). The MFI ratio was calculated by dividing the MFI of positive events by the MFI of negative events (MFI of the secondary antibody).

### 2.8. cGMP Determination

C- and GD-HUVECs were grown to confluence in six-well plates and treated as described in the experimental protocol. To stimulate NO production, both C- and GD-HUVECs were incubated with ionomycin (2 *μ*M for 24 h, Sigma-Aldrich) with or without L-NAME preincubation (1 mM for 45 min, Alexis Biochemicals, San Diego, USA). Intracellular cGMP levels were evaluated by using a commercially available Enzyme Immunoassay (EIA) kit (GE Healthcare, Little Chalfont, Buckinghamshire, UK) accordingly with the instruction provided by the supplier.

### 2.9. Statistical Analysis

The results are presented as means ± standard deviation (SD). Differences between the two cell strains and between the different treatments were analyzed by Student's *t*-test and one-way analysis of variance (ANOVA) followed by Bonferroni multiple comparison test for post hoc comparisons. The experiments were performed employing 24 cellular strains obtained from umbilical cords of 12 control and 12 GD women, respectively. Each experiment was performed using at least 3 different cellular strains (*n* = 3) and in technical duplicate or triplicate. Significance was defined as a *p* value less than 0.05.

## 3. Results

### 3.1. Effect of Ovothiol A on Endothelial Cell Viability

**Figure 1:**
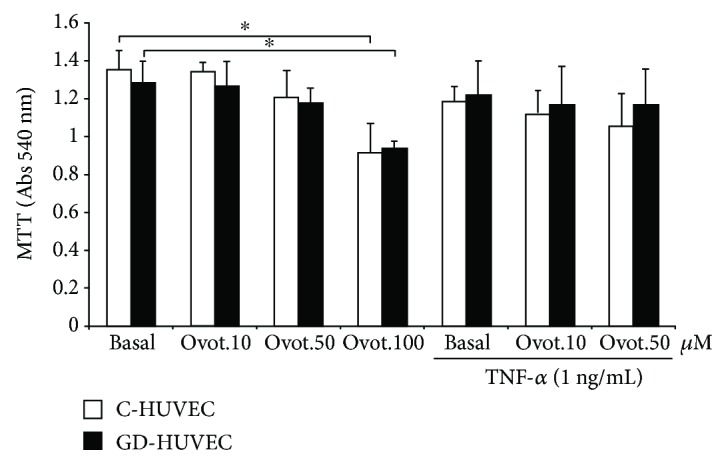
Effect of ovothiol A treatment on viability of C- and GD-HUVECs. C- and GD-HUVECs untreated (basal) and treated for 24 h with ovothiol A (10–50-100 *μ*M) with or without inflammatory stimulation by TNF-*α* (1 ng/mL) for 16 h. Data are expressed as mean ± SD, *n* = 4. ANOVA and Bonferroni multiple comparison test: ^∗^*p* < 0.05 in C- and GD-HUVECs.

The cell viability of both C- and GD-HUVECs was evaluated by the MTT assay after treatment of the cells for 24 h with different concentrations of ovothiol A. At 100 *μ*M concentration, ovothiol A was cytotoxic; thus, the maximum concentration of 50 *μ*M was used in subsequent experiments (Figure 1). Cell viability of C- and GD-HUVECs was also measured under inflammatory conditions, upon stimulation of the cells with TNF-*α* for 16 h, following treatment with ovothiol A (10 and 50 *μ*M). No significant variation in the cellular vitality was observed under inflammatory conditions (Figure 1).

### 3.2. Bioavailability of Ovothiol A in Endothelial Cells

**Figure 2:**
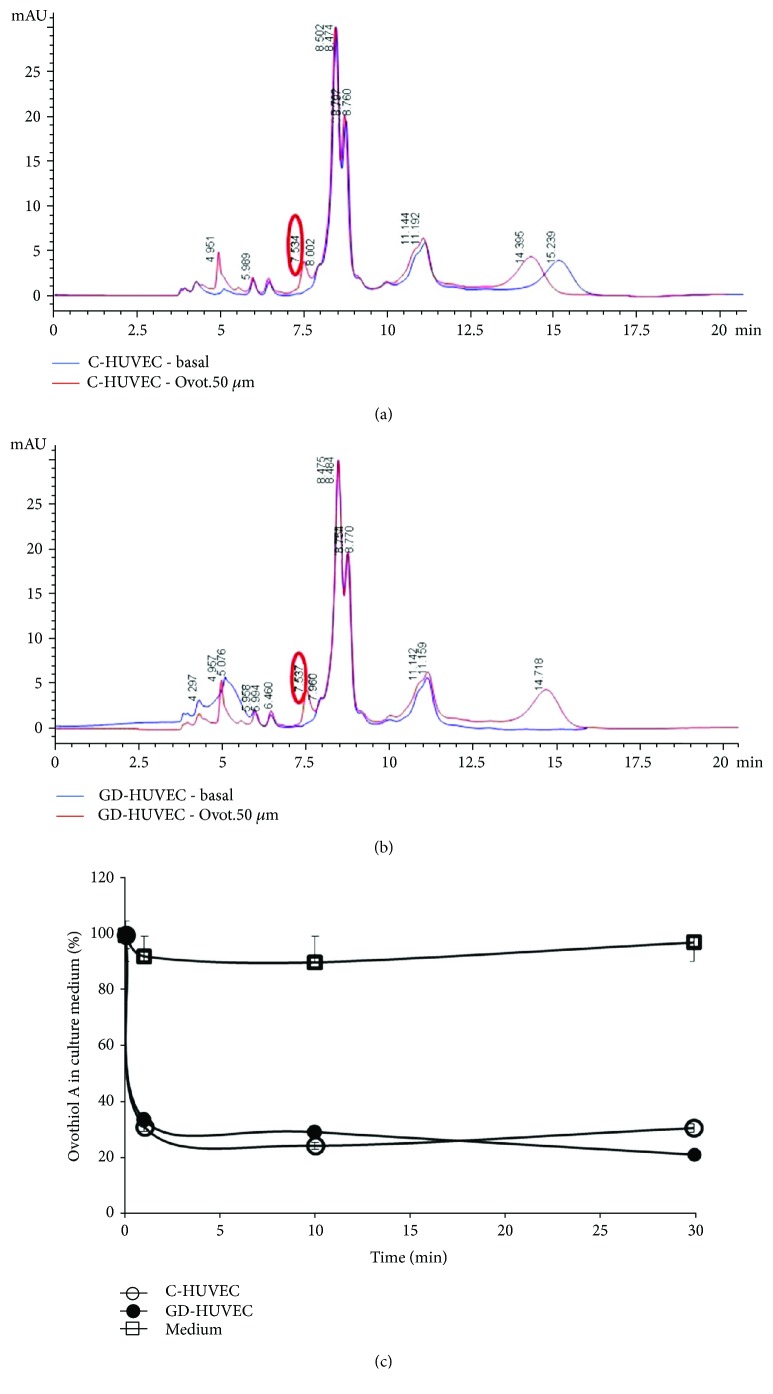
Ovothiol A levels in endothelial cells and in culture media. HPLC profiles of cytosolic extracts of C-HUVECs (a) and GD-HUVECs (b) with and without treatment with ovothiol A (50 *μ*M). Red line: C- and GD-HUVECs treated with ovothiol A for 10 min. Blue line: untreated C- and GD-HUVECs after 10 min. Detection: A280 nm. (c) Percentage of ovothiol A in the medium of C- and GD-HUVEC-treated cells.

To measure the bioavailability of ovothiol A in C- and GD-HUVECs incubated with 50 *μ*M ovothiol A, the compound was determined both inside the cells and in the culture media by HPLC analysis. The HPLC profiles of the cytosolic extracts from C-HUVECs treated with ovothiol A revealed the presence of a peak, showing the same elution time (7.5 min) of a standard solution of ovothiol A (Figure 2(a), red line). The same results were obtained for GD-HUVECs (Figure 2(b), red line). This peak was absent in untreated C- and GD-HUVECs (Figures 2(a) and 2(b), blue line). The identity of the peak eluted at 7.5 min was confirmed by LC/MS showing a pseudomolecular ion peak at *m*/*z* 401 ([M + H]^+^), corresponding to the disulphide form of ovothiol A. In some experiments, an additional peak at *m*/*z* 202 ([M + H]^+^), attributable to the reduced form of ovothiol A, was detected in GD-HUVEC. No peak at *m*/*z* 507 ([M + H]^+^), attributable to the mixed disulphide between glutathione and ovothiol, was detected. After 30 min of incubation, the levels of ovothiol A in C- and GD-HUVEC was 1.19 ± 0.02 and 1.47 ± 0.04 pmol × 10^−3^/cell, respectively. As compared to C-HUVECs, the levels of the compound inside GD-HUVEC showed a trend of increase, which did not reach statistical significance.

The decrease of ovothiol A in the medium of either cell types was very rapid, reaching 30% of the initial concentration after only 1 min (Figure 2(c)). After 30 min of incubation, the initial concentration (50 *μ*M) decreased to 14.86 ± 1.24 *μ*M and to 10.08 ± 1.68 *μ*M in the medium of C- and GD-HUVECs, respectively. Control experiments indicated that the compound was stable in the unconditioned culture medium over several hours.

### 3.3. Effect of Ovothiol A on Intracellular Glutathione Levels

**Figure 3:**
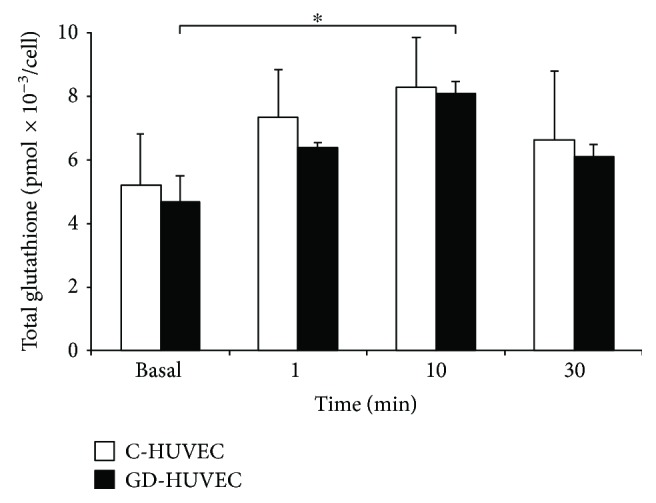
Total glutathione levels in endothelial cells. C- and GD-HUVECs untreated (basal) and treated with ovothiol A (50 *μ*M) for 1–10–30 minutes. Data are expressed as mean ± SD, *n* = 4. ANOVA and Bonferroni multiple comparison test: ^∗^*p* < 0.05 in GD-HUVECs.

In order to assess whether ovothiol A administration could affect intracellular glutathione bioavailability, total levels of this endogenous antioxidant were measured in both cellular models. In the early stages of ovothiol A treatment, the determined levels of total intracellular glutathione increased in either cell models, reaching statistical significance only in GD-HUVECs at 10 min of ovothiol A incubation (*p* < 0.05; Figure 3). Because it took place within a very short time window, whether the latter significant increase in total glutathione determinations might be accounted for by changes in glutathione biosynthesis was questioned. This has led to a checking of the possible involvement of ovothiol A in generations of these results. Control experiments revealed that ovothiol A, in its disulphide form, did not affect the glutathione assay. Indeed, standard solutions of oxidized ovothiol A did not induce any increase in TNB formation in the Glutathione Assay Kit. This result indicated that, in contrast to oxidized glutathione, oxidized ovothiol A was not a substrate for glutathione reductase present in the assay kit; indeed, in the opposite case, it would have generated a reduced ovothiol A and a resulting rise in TNB formation. On the other hand, when reduced glutathione and oxidized ovothiol were combined at a 1 to 0.5 molar ratio, a strong reduction in TNB formation was observed in comparison to the solution with the same reduced glutathione content devoid of ovothiol A disulphide, supporting in the former case a yield of a mixed disulphide, which can trap the free reduced glutathione. Once more, in the case in which the mixed ovothiol A-glutathione disulphide would have been a substrate of the glutathione reductase of the assay kit, both reduced ovothiol A and reduced glutathione would have been consequently formed and TNB formation would have been increased and not decreased. In this respect, as mentioned above, no peak attributable to the mixed disulphide between glutathione and ovothiol was found in both cell cultures, a priori also discarding its interference in the total glutathione increase mentioned above and illustrated by Figure 3. In contrast, and as mentioned above, measurements performed on cell cultures successfully detected ovothiol A in its reduced form, and this is only in GD-HUVECs. Since reduced ovothiol A, like reduced glutathione, harbours a free SH thiol function, its reaction with DTNB and its ability to increase TNB formation are not contestable. Therefore, its contribution to increased levels observed in total glutathione determinations performed by the Glutathione Assay Kit in GD-HUVECs may be considered to provide a bona fide consistent and coherent explanation though other mechanisms; for example, thiol-disulphide exchanges might also concur (for additional considerations, see Discussion).

### 3.4. Effect of Ovothiol A on Adhesion of Human Monocytes to the Endothelium

**Figure 4:**
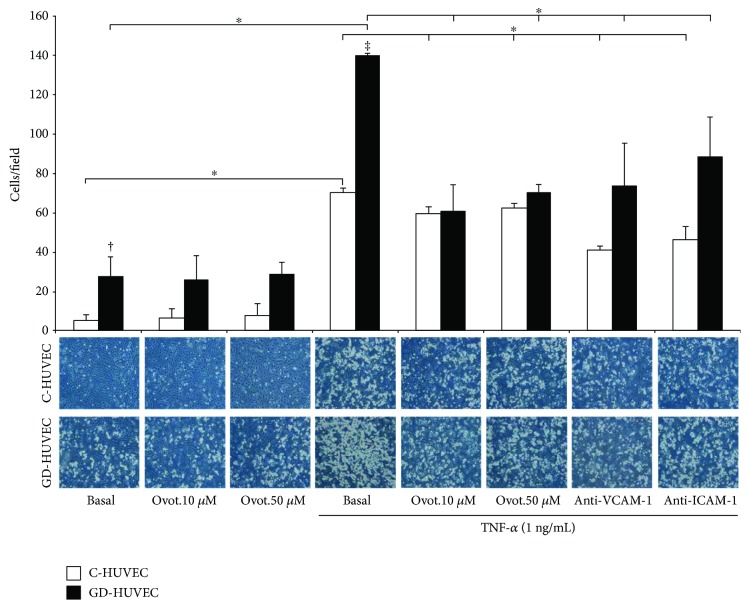
Effect of ovothiol A on TNF-*α*-induced monocyte interaction in endothelial cells. Monocyte-HUVEC adhesion in C- and GD-HUVECs untreated (basal) and incubated for 24 h with ovothiol A (10 and 50 *μ*M) and then stimulated for 16 h with or without TNF-*α* (1 ng/mL). In the histogram (upper side), quantitative data express the number of U937 cells adhering within a high-power field (3.5 mm^2^). Each measurement is expressed as mean ± SD (*n* = 3), each consisting of 8 counts per condition. Anti-VCAM-1 and anti-ICAM-1 antibody incubation, for 1 h before the assay, has been used as the negative control. In the lower side are representative photos of C- and GD-HUVECs for each experimental condition. ANOVA and Bonferroni multiple comparison test: ^∗^*p* < 0.05 in C- and GD-HUVECs. Student's *t*-test: ^†^*p* < 0.01 basal GD- versus C-HUVECs and ^‡^*p* < 0.00001 TNF-*α* GD- versus C-HUVECs.

The adhesion of the human monocytes to the endothelium was evaluated in both C- and GD-HUVECs incubated for 24 hours with 10 and 50 *μ*M ovothiol A and then stimulated with TNF-*α* (1 ng/mL) (Figure 4). As expected, the number of adherent cells on both types of HUVEC monolayers dramatically increased following the stimulation with TNF-*α*, especially in GD-HUVECs (*p* < 0.00001 versus C-HUVECs).

Interestingly, following the pretreatment with ovothiol A, a significant decrease in monocyte adhesion to C- and GD-HUVECs was observed starting from 10 *μ*M concentration (*p* < 0.05 versus TNF-*α*). Monocyte adhesion is mediated by an increased expression on the cell surface of adhesion molecules VCAM-1 and ICAM-1. Indeed, when C- and GD-HUVECs were treated with antibodies against VCAM-1 or ICAM-1 at saturating concentrations 1 hour before the assay, monocyte adhesion was suppressed in both cases (Figure 4), thus confirming that VCAM-1 and ICAM-1 hyperexpression on the cell surface plays a crucial role for increased monocyte adhesion to both HUVEC strains.

### 3.5. Effect of Ovothiol A on Membrane Exposure of Vascular Endothelial Adhesion Molecules

**Figure 5:**
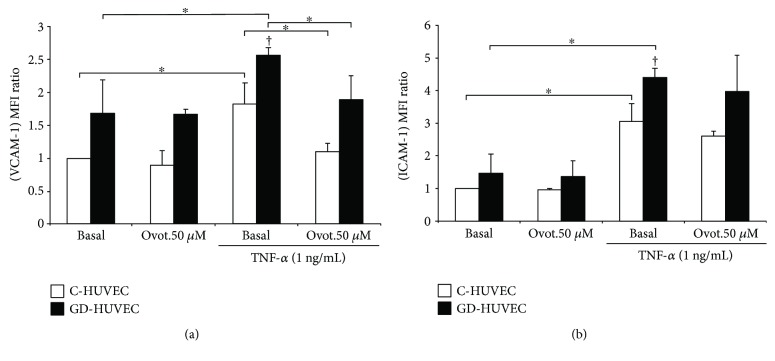
Effect of ovothiol A on adhesion molecule membrane exposure in C- and GD-HUVECs. VCAM-1 (a) and ICAM-1 (b) membrane exposure in C- and GD-HUVECs untreated (basal) and incubated for 24 h with ovothiol A (50 *μ*M) and then stimulated for 16 h with or without TNF-*α* (1 ng/mL). The results are expressed as fold increase (versus basal C-HUVECs) of the MFI ratio of surface exposure on the plasma membrane of VCAM-1 and ICAM-1 in non permeabilized cells (*n* = 3). ANOVA and Bonferroni multiple comparison test: ^∗^*p* < 0.05 in C- and GD-HUVECs. Student's *t*-test: ^†^*p* < 0.01 TNF-*α* GD- versus C-HUVECs.

Membrane exposure levels of VCAM-1 and ICAM-1 were then evaluated by flow cytometry.  Figure 5 shows increased exposure levels of VCAM-1 and ICAM-1 in both endothelial cell models after treatment with TNF-*α* (*p* < 0.05). Ovotiol A significantly reduced the exposure of VCAM-1 on the membrane in both C-HUVEC and GD-HUVEC cells (*p* < 0.05), while the effect on ICAM-1 levels did not reach statistical significance.

### 3.6. Effect of Ovothiol A on NO Bioavailability

**Figure 6:**
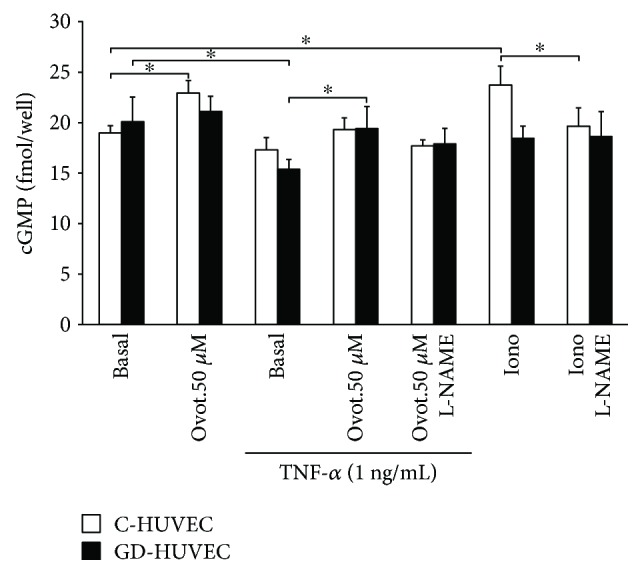
Effect of ovothiol A on intracellular cGMP levels in endothelial cells. cGMP levels measured by EIA kit in untreated (basal) and TNF-*α*-stimulated C- and GD-HUVECs after preincubation for 24 h with ovothiol A (50 *μ*M). Data are expressed as fmol/well and results by mean ± SD, *n* = 3. ANOVA and Bonferroni multiple comparison test: ^∗^*p* < 0.05 in C- and GD-HUVECs.

To better evaluate whether ovothiol A can affect NO bioavailability, we also determined cGMP levels as a proxy for the gas availability in both C- and GD-HUVECs. As shown in Figure 6, following ovothiol A treatment, basal cGMP levels significantly increased in C-HUVECs (*p* < 0.05) while it did not considerably change in GD-HUVECs. As expected, when both cell cultures were treated with TNF-*α*, cGMP levels decreased, getting statistical significance only in GD-HUVECs (*p* < 0.05). In this experimental condition, the incubation with 50 *μ*M ovothiol A slightly increased NO availability in C-HUVECs, while it was significantly augmented in GD-HUVECs (*p* < 0.05), thus indicating that ovothiol A increases NO bioavailability in these cells.

Of note, the effect of ovothiol A was not affected by preincubation with L-NAME, a known inhibitor of constitutive nitric oxide synthases (NOSs), thus suggesting that NO production is not derived from the modulation of the enzymatic activity of eNOS but by the reduction of nitro oxidative stress (Figure 6). As a positive control, ionomycin, through eNOS activation, increased cGMP levels in C-HUVECs (*p* < 0.05), which was totally abolished by L-NAME preincubation.

### 3.7. Effect of Ovothiol A on Intracellular Peroxynitrite and Superoxide Levels

**Figure 7:**
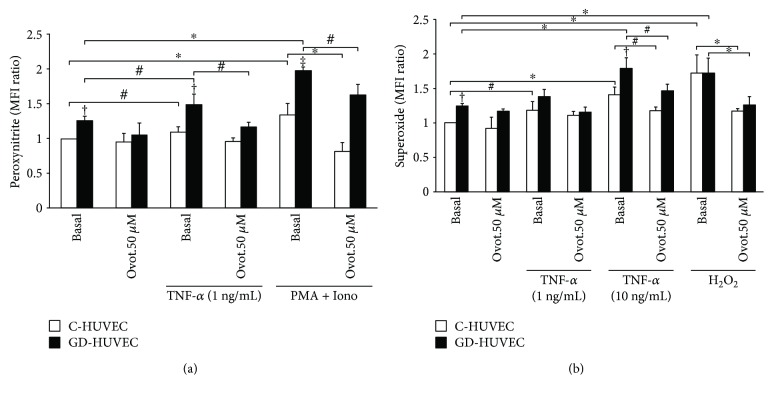
Effect of ovothiol A on peroxynitrite and superoxide anion levels in C- and GD-HUVECs. Peroxynitrite production (a) and superoxide anion levels (b) in C- and GD-HUVECs incubated for 24 h with ovothiol A (50 *μ*M) with or without TNF-*α*-stimulation (1 and 10 ng/mL) for 16 h. PMA (200 ng/mL) + Iono (50 nM) and H_2_O_2_ (300 *μ*M) for 30 min before the assay are used as positive controls for endogenous peroxynitrite and superoxide anion production, respectively. Data are expressed as the MFI ratio (compared to basal C-HUVECs) of 4 independent experiments (*n* = 4). ANOVA and Bonferroni multiple comparison test: ^∗^*p* < 0.05 in C- and GD-HUVECs. Student's *t*-test: ^#^*p* < 0.05 in C- and GD-HUVECs, ^†^*p* < 0.01 basal and TNF-*α* GD- versus C-HUVECs, and ^‡^*p* < 0.001 PMA + Iono GD- versus C-HUVECs.

Following TNF-*α* stimulation, the levels of peroxynitrite (a marker of nitro oxidative stress) significantly increased in both C- and GD-HUVECs (*p* < 0.05). However, the level of peroxynitrite was significantly greater in GD-HUVECs compared to C-HUVECs, both in basal condition and following TNF-*α* stimulation (*p* < 0.01), confirming that GD-HUVECs exhibit a greater basal O_2_^•−^ generation together with increased NO levels [28]. In this experimental condition, 50 *μ*M ovothiol A significantly reduced the levels of TNF-*α*-induced peroxynitrite only in GD-HUVECs (*p* < 0.05), suggesting that in this cellular model ovothiol A can downregulate the TNF-*α*-increased nitro oxidative stress. As positive control, ionomycin in combination with phorbol 12-myristate 13-acetate (PMA) induced peroxynitrite formation in both HUVEC cells, but especially in GD-HUVECs (*p* < 0.001 versus C-HUVECs). Notably, ovothiol A preincubation significantly reduced the levels of peroxynitrite in both cultures (Figure 7(a)).

As shown in Figure 7(b), the basal level of superoxide anion was significantly higher in GD- compared to C-HUVECs (*p* < 0.01). The treatment with 1 ng/mL TNF-*α* induced a significant superoxide formation in C-HUVECs while the dose of 10 ng/mL increased superoxide levels in both C- and GD-HUVECs (*p* < 0.05). Interestingly, ovothiol A totally reversed the 10 ng/mL TNF-*α* effect in both HUVEC cultures. The same results were observed after H_2_O_2_ treatment (positive control).

## 4. Discussion

Ovothiol A derived from marine edible sources, such as sea urchin eggs, sea cucumbers, and oysters, may represent a promising marine bioactive molecule for pharmaceutical and nutraceutical applications. In particular, the eggs from sea urchin *Paracentrotus lividus* have been regarded as a culinary delicacy since ancient times. One of the peculiar features of these eggs is the presence of ovothiol A at millimolar concentrations. However, this abundant source of antioxidant compound has not been fully appreciated and exploited. Recently, we have discovered that ovothiol A, purified from the eggs of the sea urchin *P. lividus* [8, 9], exhibits an antiproliferative activity in hepatocarcinoma cell lines through an autophagic mechanism, not involving scavenging of reactive oxygen species (ROS) [27]. Conversely, the neuroprotective activity of an ovothiol analogue, which regulates redox homeostasis, has been previously documented by Vamecq et al. [26].

Based on these results on the biological activities of ovothiols in mammalian model systems, the synthesis of this class of compounds has been reconsidered, leading to a good amount of unmethylated precursors of ovothiols, 5-thiohistidines [33], thus opening new perspectives in the potential use of this molecule as a new drug of marine origin.

Stimulated by the increasing interest in new natural molecules that can exert cardiovascular protective effects, we have investigated for the first time the biological activities of ovothiol A, isolated from sea urchin eggs of *P. lividus*, in an *in vitro* model of cultured endothelial cells obtained from umbilical cords of gestational diabetic mothers and control women. Interestingly, we have previously demonstrated that these endothelial cells exposed even transiently to *in vivo* hyperglycemia, oxidative stress, and inflammation exhibit durable proatherogenic modifications characterized by reduced bioavailability of nitric oxide (NO), thus mimicking endothelial dysfunction associated with diabetes [28]. Moreover, in our earlier study, we determined in C-HUVECs a significant increase in the generation of ROS and nitrotyrosine (index of peroxynitrite formation) and a parallel reduction of NO biological activities following TNF-*α* treatment [2]. The attenuation of NO bioavailability was documented by cGMP-decreased levels, a good proxy for the evaluation of NO availability because in normal conditions soluble guanylate cyclase is activated by nanomolar concentrations of gas with a subsequent increase in cGMP content [34]. Therefore, all these features make this cellular model particularly useful for the evaluation of natural molecules that can play a potential protective role in vascular homeostasis [28].

In the present study, employing both endothelial strains, we have found that ovothiol A, in its disulphide form at the non toxic concentration of 50 *μ*M, induced a significant vascular antioxidant/anti-inflammatory effect. In particular, we have demonstrated that, under TNF-*α*-induced proinflammatory conditions, ovothiol A (10 and 50 *μ*M) induces a significant decrease in monocyte adhesion into both C- and GD-HUVECs and this is associated with the reduced exposure of VCAM-1 on the endothelium membrane surface (Figures 4 and 5(a)). It is not surprising that the expression of ICAM-1 is only moderately reduced by ovothiol A (Figure 5(b)) since this endothelial adhesion molecule is characterized by a constitutive expression which is slightly modulated by exogenous stimuli [28].

To better support the observed anti-inflammatory activity of ovothiol A, we first evaluated the intracellular bioavailability of the molecule and its capability to regulate intracellular glutathione levels in this model system. As shown in Figure 2(c), ovothiol A is rapidly absorbed by both control and GD-HUVECs up to 70% of the initial concentration, suggesting that 50 *μ*M may represent a saturating concentration. Indeed, the anti-inflammatory effects of the compound on these cells are also appreciable at levels as low as 10 *μ*M (Figure 4).

In both cell strains, ovothiol A was found largely in its disulphide form, as it was added to the culture medium (Figure 2). However, we cannot rule out that under reductive conditions of the cellular environment, ovothiol A administered in the form of disulphide may be partially reduced by the GSH/GSSG system and then rapidly react with other cellular thiols, such as cysteine residues exposed on the surface of proteins to form mixed disulphide bridges [35, 36].

Indeed, we were able to detect ovothiol A in its reduced form only in GD-HUVECs, in which increased intracellular glutathione levels were observed following 10 min of incubation (Figure 3). These results suggest that when entering the cells in its disulphide form, ovothiol is partially reduced, likely by GSH, and it can both react with ROS and compete with other mixed disulphides in the cells, such as those arising from bonding of glutathione with cysteine residues of proteins (protein glutathionylation). This could account for the increase in free total glutathione observed after 10 min in GD-HUVECs. This hypothesis is also supported by the fact that under oxidative stress conditions, such as those of GD-HUVECs [28], the proportion of GSH versus GSSG increases in favour of GSSG (preliminary data not shown), which in this form can react with the exposed cysteines of proteins to give mixed disulphides to protect them from further oxidation [35, 36].

Notably, following stimulation of both cell strains with TNF-*α*, the superoxide increased mainly in GD-HUVECs, thus supporting the idea that higher levels of reduced GSH in control endothelial cells could buffer cytokine-induced superoxide increase. As a result, under proinflammatory conditions, the ability of ovothiol A to reduce nitro oxidative stress is observed mainly in GD-HUVECs (Figure 7).

As mentioned above, one of the most important roles in the maintenance of vascular homeostasis is played by NO bioavailability. Able to exert its anti-inflammatory effect through the downregulation of vascular adhesion molecule membrane exposure, this gas can react very easily with high concentrations of superoxide anion giving rise to peroxynitrite [3]. This mechanism, which under inflammatory stimuli leads to a greater reduction of NO bioavailability in GD-HUVEC (indirectly evaluated by cGMP levels as shown in Figure 6), is associated with a parallel increase in peroxynitrite levels (Figure 7(a)). Notably, we observed a decrease in peroxynitrite production after addition of ovothiol A especially in GD-HUVECs, confirming the proposed antioxidant activity of the molecule (Figure 7(a)). Indeed, the finding that the shown ovothiol A activity was not affected by preincubation with L-NAME, the inhibitor of constitutive isoforms of NOS, suggested that the increased bioavailability of NO does not result from the increase of eNOS enzymatic activity but by the ovothiol A-induced lowering of oxidative stress. Moreover, the increased availability of NO induced by ovothiol A is associated with a significant reduction of TNF-*α*-stimulated lymphomonocyte adhesion to both endothelial cell cultures (Figure 4).

Overall, these events significantly support the idea that this marine molecule might play an important role in inhibiting the mechanisms leading to chronic endothelial low-grade inflammation also under physiopathological conditions, as chronic hyperglycemia, largely associated with increased cardiovascular events. Thus, ovothiol A action *in vivo* might actually result in the reduction of atherosclerotic vascular modifications.

## 5. Conclusion and Perspective

In summary, ovothiol A in its disulphide form exerts antioxidant and anti-inflammatory activities in the cellular model employed in our study, and in particular, this action is more effective in cells mimicking vascular diabetic conditions. This effect involves a reduction of reactive oxygen and nitrogen species and an increase in NO bioavailability.

When comparing the effects and the doses of ovothiol used in this study with those observed for other known antioxidant/anti-inflammatory natural molecules, we can conclude that in our cellular model the effective concentration of ovothiol A (10–50 *μ*M) is comparable to that of carotenoids often used as supplements for this pathology [2]. Moreover, the ovothiol A concentrations used in this study were lower compared to other compounds, such as lipoic acid, commonly used as drug for the treatment of diabetes at concentrations of 100–200 *μ*M [1]. Interestingly, also ergothioneine, a 2-thiohistidine, produced by fungi and cyanobacteria, has been recently shown to have cardiovascular therapeutic potential [37].

Therefore, the results of this study open new perspectives on the potential of ovothiol as a dietary supplement or a drug to prevent and/or treat chronic low-grade inflammation associated with the development of atherosclerotic processes and cardiovascular diseases, particularly in diabetes.

## Figures and Tables

**Table 1:** Clinical characteristics of control (C, *n* = 12) and gestational diabetic (GD, *n* = 12) women.

Characteristic	C women	GD women
Age (years)	35.83 ± 6.69	34.5 ± 6.02
Height (cm)	163.29 ± 5.82	160.5 ± 8.28
Pregestational weight (kg)	67.11 ± 12.37	65.83 ± 10.19
BMI (kg/m^2^)	27.25 ± 4.74	28.39 ± 2.61
OGTT values (mmol/L)		
Basal glycaemia	4.56 ± 0.26	5.06 ± 0.27^∗^
1 h glycaemia	8.13 ± 0.91	10.36 ± 1.1^#^
2 h glycaemia	6.37 ± 1.11	8.17 ± 1.38^∗^
OGTT gestational week	28 ± 2.44	24.33 ± 4.59
SBP (mm/Hg)	108 ± 8	102.4 ± 9.17
DBP (mm/Hg)	70.33 ± 8.52	65.6 ± 10.73

Data are expressed as mean ± SD. BMI: body mass index; OGTT: oral glucose tolerance test; SBP: systolic blood pressure; BDP: diastolic blood pressure. ^∗^*p* < 0.002 and ^#^*p* < 0.0001.
